# A deep learning approach for fully automated cardiac shape modeling in tetralogy of Fallot

**DOI:** 10.1186/s12968-023-00924-1

**Published:** 2023-02-27

**Authors:** Sachin Govil, Brendan T. Crabb, Yu Deng, Laura Dal Toso, Esther Puyol-Antón, Kuberan Pushparajah, Sanjeet Hegde, James C. Perry, Jeffrey H. Omens, Albert Hsiao, Alistair A. Young, Andrew D. McCulloch

**Affiliations:** 1 Department of Bioengineering, University of California San Diego, 9500 Gilman Drive, MC 0412, La Jolla, CA 92093-0412 USA; 2 Department of Biomedical Engineering, King’s College London, London, UK; 3 Department of Pediatrics, University of California San Diego, La Jolla, CA USA; 4 Division of Cardiology, Rady Children’s Hospital San Diego, San Diego, CA USA; 5 Department of Radiology, University of California San Diego, La Jolla, CA USA

**Keywords:** Cardiovascular magnetic resonance (CMR), Image segmentation, Deep learning, Shape modeling, Congenital heart disease

## Abstract

**Background:**

Cardiac shape modeling is a useful computational tool that has provided quantitative insights into the mechanisms underlying dysfunction in heart disease. The manual input and time required to make cardiac shape models, however, limits their clinical utility. Here we present an end-to-end pipeline that uses deep learning for automated view classification, slice selection, phase selection, anatomical landmark localization, and myocardial image segmentation for the automated generation of three-dimensional, biventricular shape models. With this approach, we aim to make cardiac shape modeling a more robust and broadly applicable tool that has processing times consistent with clinical workflows.

**Methods:**

Cardiovascular magnetic resonance (CMR) images from a cohort of 123 patients with repaired tetralogy of Fallot (rTOF) from two internal sites were used to train and validate each step in the automated pipeline. The complete automated pipeline was tested using CMR images from a cohort of 12 rTOF patients from an internal site and 18 rTOF patients from an external site. Manually and automatically generated shape models from the test set were compared using Euclidean projection distances, global ventricular measurements, and atlas-based shape mode scores.

**Results:**

The mean absolute error (MAE) between manually and automatically generated shape models in the test set was similar to the voxel resolution of the original CMR images for end-diastolic models (MAE = 1.9 ± 0.5 mm) and end-systolic models (MAE = 2.1 ± 0.7 mm). Global ventricular measurements computed from automated models were in good agreement with those computed from manual models. The average mean absolute difference in shape mode Z-score between manually and automatically generated models was 0.5 standard deviations for the first 20 modes of a reference statistical shape atlas.

**Conclusions:**

Using deep learning, accurate three-dimensional, biventricular shape models can be reliably created. This fully automated end-to-end approach dramatically reduces the manual input required to create shape models, thereby enabling the rapid analysis of large-scale datasets and the potential to deploy statistical atlas-based analyses in point-of-care clinical settings. Training data and networks are available from cardiacatlas.org.

## Background

Advances in computational medicine have enabled more quantitative approaches to characterizing ventricular shape and remodeling in individuals with heart disease. One such approach is the use of cardiac shape modeling to condense complex, multi-dimensional data from standard of care cardiovascular magnetic resonance (CMR) images into statistical atlases of cardiac structure and function [1–13]. These atlases are composed of interpretable shape and wall motion features that can be important quantitative biomarkers of patient status and outcome and, in turn, aid in prognosis and treatment of disease.

**Fig. 1:**
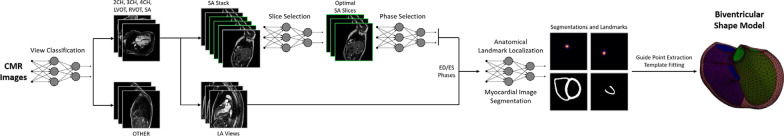
Overview of the automated cardiac shape modeling pipeline. The automated pipeline was developed as a series of five steps for view classification, slice selection, phase selection, anatomical landmark localization, and myocardial image segmentation. *CMR* cardiovascular magnetic resonance, *2Ch* two-chamber, *3Ch* three-chamber, *4Ch* four-chamber, *LVOT* left ventricular outflow tract, *RVOT* right ventricular outflow tract, *SAx* short axis, *LA* long axis, *ED* end-diastole, *ES* end-systole

To extract the relevant features of cardiac morphology that are used to build these statistical atlases, several steps are involved (Fig. 1). Traditionally, most of these have been performed manually, requiring a human analyst to identify relevant view and slice information from a raw CMR image dataset, identify end-diastolic (ED) and end-systolic (ES) phases in the cardiac cycle, label anatomical features such as the left ventricular (LV) apex and valvular insertion points, and trace endocardial and epicardial contours. This information can then be collated and processed to build three-dimensional (3D), biventricular shape models, including all four valves (aortic, pulmonary, mitral, tricuspid), and used to build atlases of ED, ES, or systolic wall motion (ES-ED) using principal component analysis. Semi-automated methods for image segmentation have been developed that take advantage of guide-point modeling [14–17], and more recent efforts have focused on using deep learning (e.g., convolutional neural networks (CNNs), fully convolutional neural networks (FCNs), U-nets, and recurrent neural networks (RNNs)) to completely automate image segmentation [18, 19]. Fully manual and even semi-automated techniques, however, are time-consuming and require significant operator expertise to achieve an acceptable level of accuracy. While fully automated methods have made advances in accuracy, they are prone to error for challenging regions of the heart such as the right ventricle (RV) and the complex anatomies of congenital heart disease (CHD) patients.

With improved availability of large, heterogenous clinical datasets and manually annotated models for reference, the major steps involved in constructing 3D, biventricular shape models from raw CMR image datasets for use in statistical atlas-based analyses can be automated. Herein, we detail the use of deep learning for automated view classification, slice selection, phase selection, anatomical landmark localization, and myocardial image segmentation that together provide an end-to-end pipeline for cardiac shape modeling. Moreover, we demonstrate this approach in a multi-institutional, international cohort of patients with repaired tetralogy of Fallot (rTOF)—a patient population with particularly challenging anatomy. The integration of these steps in an automated fashion can significantly reduce the manual input and time required to create shape models, which has been a significant barrier to the clinical application of atlas-based analyses to patient management.

## Methods

### Study population and data acquisition

**Fig. 2:**
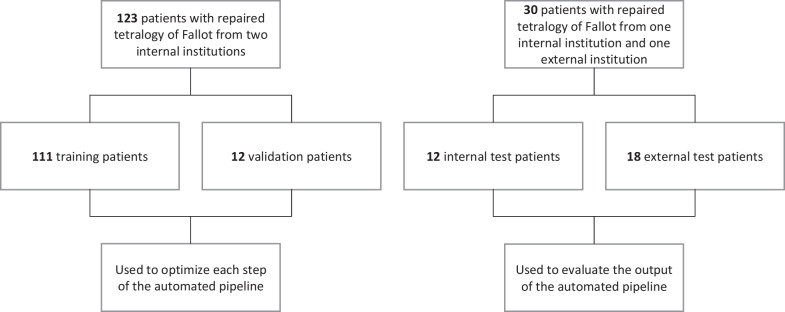
Flow-diagram of internal and external datasets used to train, validate, and test the automated cardiac shape modeling pipeline. Cases from the training/validation set were used to optimize each step of the automated pipeline, while cases from the test set were used to evaluate the generalizability of the automated pipeline

This study used deidentified, retrospective CMR images of patients with rTOF from three clinical centers (Rady Children’s Hospital, San Diego, California, USA; The Center for Advanced Magnetic Resonance Imaging, Auckland, NZ; and Evelina Children’s Hospital, London, UK) with approval from local institutional review boards via waiver of informed consent (UCSD IRB 201,138; HDEC 16/STH/248; and 21/LO/0650, respectively). Labeled CMR images from 123 rTOF patients were contributed from the Cardiac Atlas Project (CAP) database (https://www.cardiacatlas.org) [20] from San Diego and Auckland (internal sites) and were used as the training/validation set to optimize each step in the automated pipeline. A separate test set composed of labeled CMR images from 30 rTOF patients from San Diego (internal site) and London (external site) was used to evaluate the output of the automated pipeline. A flow-diagram summarizing the datasets employed and how they were used to develop the automated pipeline is shown in Fig. 2. Summary characteristics of the study participants in the training/validation and test sets are shown in Table 1. All patients underwent functional CMR examination within the scope of standard clinical practice. CMR acquisition data for study participants in the training/validation and test sets are shown in Table 2.

**Table 1:** Summary characteristics of study participants in the training/validation and test sets

Characteristic	Training/validation set (n = 123)	Test set (n = 30)	*p*-value
Sex (m/f)	73/50	15/15	0.35
Age (y)	17 (12–26)	22 ± 13	0.47
Height (cm)	161 (150–168)	163 (155–176)	0.07
Weight (kg)	58 ± 25	63 ± 18	0.31
BSA (m^2^)	1.57 ± 0.42	1.72 (1.52–1.82)	0.27
LV EDV (mL)	128 ± 44	119 ± 36	0.31
LV ESV (mL)	66 (51–83)	60 (47–70)	0.19
LV SV (mL)	59 ± 21	57 ± 16	0.52
LV EF (%)	48 (41–52)	48 ± 7	0.23
LV mass (g)	118 ± 35	111 ± 33	0.28
RV EDV (mL)	205 ± 67	197 ± 51	0.54
RV ESV (mL)	127 ± 46	121 ± 37	0.48
RV SV (mL)	78 ± 28	76 ± 23	0.78
RV EF (%)	38 ± 7	39 ± 7	0.72
RV mass (g)	58 (43–77)	53 ± 24	0.16

Data are reported as mean ± standard deviation or as median (interquartile range), depending on the distribution, for continuous variables and as the count for categorical variables. Normality was tested using Shapiro-Wilks. Differences between the training/validation set and test set were assessed using two-sample t-tests or Wilcoxon rank-sum tests, depending on the distribution, for continuous variables and Pearson’s chi-squared tests for categorical variables. *BSA* body surface area, *LV* left ventricular; *RV* right ventricular, *EDV* end-diastolic volume, *ESV* end-systolic volume, *SV* stroke volume, *EF* ejection fraction

**Table 2:** CMR acquisition data for study participants in the training/validation and test sets

Parameter	Training/validation set (n = 123)	Test set (n = 30)
Imaging		
Flip angle (°)	64 (15–80)	57 (45–81)
Phase spatial resolution (mm)	1.9 (0.9–3.0)	1.4 (0.5–2.1)
Frequency spatial resolution (mm)	1.6 (0.8–2.8)	1.4 (0.5–2.1)
Slice thickness (mm)	7.1 (4.0–10.0)	7.7 (4.5–10.0)
Repetition time (ms)	15.8 (2.6–48.7)	16.6 (2.7–60.5)
Echo time (ms)	1.4 (1.1–3.3)	1.4 (1.1–1.8)
Magnetic field strength		
1.5T	120 (98)	30 (100)
3T	3 (2)	–
Manufacturer		
Siemens Healthineers	55 (45)	13 (43)
Philips Healthcare	41 (33)	8 (27)
GE Healthcare	27 (22)	9 (30)
Model		
Siemens Avanto	55 (45)	3 (10)
Siemens Intera	41 (33)	8 (27)
GE Discovery MR450	14 (11)	3 (10)
GE Signa HDxt	10 (9)	1 (3)
GE Discovery MR750w	3 (2)	–
Siemens Aera	–	10 (33)
Philips Achieva	–	3 (10)
Philips Ingenia	–	2 (7)

Numerical data are reported as mean (range). Categorical data are reported as the count (percentage). *CMR* cardiovascular magnetic resonance

### Automated cardiac shape modeling pipeline overview

The automated cardiac shape modeling pipeline was developed as a series of five steps for view classification, slice selection, phase selection, anatomical landmark localization, and myocardial image segmentation, respectively. The view classification network was designed to take a raw CMR image dataset and classify views as either two-chamber left (2Ch LT), two-chamber right (2Ch RT), three-chamber (3Ch), four-chamber (4Ch), LV outflow tract (LVOT), RV outflow tract (RVOT), short axis (SAx), or other. After view classification, optimal and non-optimal slices in the SAx stack were characterized through the slice selection network. Optimal slices were defined as SAx slices that range from the LV apex to the mitral and tricuspid base planes, while non-optimal slices were defined as SAx slices either below the LV apex or above the mitral and tricuspid base planes. ED and ES phases were then identified from selected SAx slices through the phase selection network. ED and ES phases from the 3Ch, 4Ch, RVOT, and selected SAx slices were then provided as inputs to the anatomical landmark localization networks to identify the LV apex, RV inserts, and mitral, tricuspid, aortic, and pulmonary valve inserts on corresponding views. These anatomical landmarks are required for use with previously developed mesh fitting software, as described below. Finally, ED and ES phases from the 2CH LT, 2CH RT, 3Ch, 4Ch, RV outflow tract (RVOT), and selected SAx slices were segmented using the myocardial image segmentation network from which contour points were extracted for the LV and RV endocardium, epicardium, and septum. The LV papillary muscles and RV trabeculae were included in the blood pool. The extracted contour points and the anatomical landmark points were then converted from image to model coordinates using an affine transformation and fit to a previously developed biventricular subdivision surface template mesh [21, 22] via diffeomorphic non-rigid registration for contour points and landmark registration for anatomical landmark points. An overview of the automated cardiac shape modeling pipeline is detailed in Fig. 1. Each step in the pipeline was designed to give the user the ability to make manual corrections if necessary.

### Technical specifications, network architectures, and optimization

For each step in the automated pipeline (view classification, slice selection, phase selection, anatomical landmark localization, and myocardial image segmentation), we report technical specifications regarding the dataset and preprocessing, network architecture, and optimization and evaluation. For the development of the view classification, slice selection, phase selection, and anatomical landmark localization networks, we utilized Python (v3.6.15, Python Software Foundation, Wilmington, Delaware, USA) and Tensorflow v2.4 on a machine with an NVIDIA Tesla V100 GPU. For myocardial image segmentation, we utilized Python v3.7.10 and PyTorch v1.8.1 on a machine with an NVIDIA GeForce RTX 3090 GPU. The 123 cases from the CAP database (https://www.cardiacatlas.org) were randomly split at the patient level into 111 training and 12 validation cases (90–10 percent split), with roughly equal cases from each internal site, San Diego and Auckland, in each set (Fig. 2). For each network detailed below, training cases with appropriate data were used to optimize the network weights, while validation cases with appropriate data were used for hyper parameter tuning and to estimate model performance.

### View classification

#### Dataset and preprocessing

Of the 111 cases in the training set, 93 had complete CMR studies available (n = 18 excluded) and were included in the training of the view classification network. Similarly, 8 of the cases in the validation set had complete raw CMR studies available (n = 4 excluded) and were used for validation. Each CMR series was manually classified into one of eight possible view categories: 2Ch LT, 2Ch RT, 3Ch, 4Ch, LVOT, RVOT, SAx, or other. Prior to training, each CMR image was converted to an 8-bit integer RGB image and resized to 224 × 224 pixels using bicubic interpolation. Images were normalized by zero-centering each color channel with respect to the ImageNet dataset, without scaling. To improve model generalizability, real-time data augmentations were utilized during training including random rotations (± 10%), random zooms (± 20%), and random translations (± 10%).

#### Network architecture

For view classification, the CNN architecture ResNet50 was utilized. Feature extraction layers were imported with pretrained weights from the ImageNet dataset. Classification layers consisted of a 2D global average pooling layer followed by a fully connected dense layer with eight output classes and softmax activation.

#### Optimization and evaluation

Prior to training, the pretrained weights in the feature extraction layers were frozen. The classification layers were then optimized with a sparse categorical cross entropy loss function for a total of 25 epochs using a batch size of 16 and a stochastic gradient descent optimizer with a learning rate of 0.0001 and momentum of 0.9. Next, the feature extraction layer weights were unfrozen, the learning rate was decreased by a factor of 2, and training was continued for an additional 50 epochs. Following training, view classification performance was assessed using precision, recall, and F1-scores.

### Slice selection

#### Dataset and preprocessing

All 111 cases in the training set and all 12 cases in the validation set had available SAx stacks and were included for the optimization of a SAx slice selection network. SAx slices were split into two possible classifications: optimal and non-optimal. Optimal slices were defined as slices that were manually selected for inclusion in the modeling process by users, which typically range from the LV apex to the mitral and tricuspid base planes. Non-optimal slices were defined as slices that were not included in the modeling process by the manual users. Of note, not every slice between the apex and valve planes is required for modeling; as a result, there was considerable variability in which slices were selected as optimal between cases and users. Prior to training, each CMR image was converted to an 8-bit integer RGB image and resized to 224 × 224 pixels using bicubic interpolation. Images were normalized by zero-centering each color channel with respect to the ImageNet dataset, without scaling. To improve model generalizability, real-time data augmentations were utilized during training including random rotations (± 30%), random zooms (± 20%), and random translations (± 10%).

#### Network architecture

For slice selection, the CNN architecture ResNet50 was utilized. Feature extraction layers were imported with pretrained weights from the ImageNet dataset. Classification layers consisted of a 2D global average pooling layer followed by a fully connected dense layer with eight output classes and softmax activation.

#### Optimization and evaluation

Prior to training, the pretrained weights in the feature extraction layers were frozen. The classification layers were then optimized with a sparse categorical cross entropy loss function for a total of 25 epochs using a batch size of 16 and a stochastic gradient descent optimizer with a learning rate of 0.0001 and momentum of 0.9. Next, the feature extraction layer weights were unfrozen, the learning rate was decreased by a factor of 2, and training was continued for an additional 50 epochs. Following training, slice selection performance was assessed using precision, recall, and F1-scores.

### Phase selection

#### Dataset and preprocessing

All 111 cases in the training set and all 12 cases in the validation set were used to optimize the phase selection network. To produce ground-truth labels, the ES phase was manually labeled for each case using a mid-ventricular slice from the SAx stack. The ES phase was determined using the LV and defined as the phase when the LV cavity volume was at a minimum. This label was used to produce a normalized Gaussian distribution centered at the ES phase, with a sigma of 4. In this way, a numerical value was assigned to each phase of the cardiac cycle, increasing to 1 during systole and decreasing to 0 during diastole.

Inputs consisted of SAx slices ranging from apex to base. For each slice, CMR images from the complete cardiac cycle were utilized, producing a 2D + time input with 30 phases. Cases with less than 30 phases in the SAx stack were zero-padded to maintain a consistent input size. Prior to training, each CMR image was converted to an 8-bit integer RGB image and resized to 224 × 224 pixels using bicubic interpolation. Images were normalized by zero-centering each color channel with respect to the ImageNet dataset, without scaling. To improve model generalizability, real-time data augmentations were utilized during training, including resizing to 256 × 256 pixels and randomly cropping back to 224 × 224 pixels, random brightness adjustments (± 10%), and random contrast adjustments (± 5%). Inputs were also randomly shuffled along the time axis, such that the ground-truth ES phase could occur at any time point in the 30-phase input.

#### Network architecture

The phase selection network consisted of a CNN combined sequentially with a long short-term memory (LSTM) network. This network was chosen based on previously published cardiac phase selection networks [23, 24]. The CNN is used to extract image features, while the LSTM encodes temporal information. For the CNN feature extractor, the CNN architecture ResNet50 was utilized. Feature extraction layers were imported with pretrained weights from the ImageNet dataset. The ResNet50 architecture was followed by two LSTM layers and two fully connected dense layers.

#### Optimization and evaluation

Prior to training, the pretrained weights in the feature extraction layers were frozen. The LSTM layers were then optimized with a mean squared error loss function for a total of 75 epochs using a batch size of 4 and a stochastic gradient descent optimizer with a learning rate of 0.0005 and momentum of 0.9. Next, the feature extraction layer weights were unfrozen and training was continued for an additional 150 epochs. Following training, ES phase selection performance was assessed using the average absolute frame difference (AAFD) between predictions and manual labels.

### Anatomical landmark localization

#### Dataset and preprocessing

All 111 cases in the training set and all 12 cases in the validation set were used to optimize the anatomical landmark localization networks. From these cases, the 3Ch, 4Ch, RVOT, and optimal SAx slices were selected. Ground truth anatomical landmarks were manually placed throughout the cardiac cycle for each view by an expert analyst using Cardiac Image Modeller (CIM) software (Auckland, NZ) [25]. In the 3Ch view, mitral valve inserts and aortic valve inserts were labeled. In the 4Ch view, mitral valve inserts, tricuspid valve inserts, and the LV apex were labeled. In the RVOT view, pulmonary valve inserts were labeled. In the SAx slices, RV inserts were labeled. Manual point labels were converted to a normalized Gaussian heat map label with a sigma of 12 for all images. Gaussian heat maps were utilized based on recently published literature on cardiac landmark localization [26].

For each cardiac view, inputs consisted of 2D images throughout the cardiac cycle. To provide temporal information, the input for each time point t was concatenated with 2D images from t-2, t-1, t + 1, and t + 2, producing a final 2D + time input with 5 channels. Prior to training, the inputs were resized to 256 × 256 pixels using bicubic interpolation and normalized to have a minimum of 0 and maximum of 1. To improve model generalizability, real-time data augmentations were utilized during training, including random rotations (± 10%), random zooms (± 20%), random translations (± 10%), random contrast adjustments (± 15%), the addition of Gaussian noise, and histogram equalizations.

#### Network architecture

The anatomical landmark localization networks utilized the U-net architecture, an encoder-decoder with skip connections between mirrored layers in the encoder and decoder stacks [27]. Scaled exponential linear units (SELU) were utilized for activation, with a LeCun normal kernel initializer [28]. An individual U-net network was optimized for each cardiac view, with the number of output channels determined by the number of landmarks present in each view.

#### Optimization and evaluation

For each cardiac view, a U-net network was optimized with a mean squared error loss function for a total of 150 epochs using a batch size of 40 and a stochastic gradient descent optimizer with a learning rate of 1e-5 and momentum of 0.9. Following training, performance for each network was assessed using absolute distance errors between predicted and ground truth landmarks. For insertion points, the angulation error between predicted and ground truth valve and septal planes was also measured.

### Myocardial image segmentation

#### Dataset and preprocessing

All 111 cases in the training set and all 12 cases in the validation set were used to optimize the myocardial image segmentation networks. From these cases, the 2Ch LT, 2Ch RT, 3Ch, 4Ch, RVOT, and optimal SAx slices were selected. Ground truth myocardial image segmentations were generated from contours that were manually drawn at ED and ES for each view by an expert analyst with greater than 10 years of cardiac modeling experience using Segment (Medviso, Lund, Sweden) [29]. The LV papillary muscles and RV trabeculae were included in the blood pool. In the 2Ch LT view, the LV cavity and LV myocardium were labeled. In the 2Ch RT and RVOT views, the RV cavity and RV myocardium were labeled. In the 3Ch, 4Ch, and SAx views, the LV/RV cavity and LV/RV myocardium were labeled.

For each cardiac view, inputs consisted of 2D images at ED and ES. Prior to training, inputs were cropped to their non-zero regions and normalized to have a minimum of 0 and maximum of 1. To improve model generalizability, real-time data augmentations were utilized during training, including random rotations (± 10%), random zooms (± 20%), random brightness and contrast adjustments (± 15%), the addition of Gaussian noise and blur, gamma correction, mirroring, and the simulation of low resolution.

#### Network architecture

The myocardial image segmentation networks utilized the nnU-net architecture, an encoder-decoder with skip connections between mirrored layers in the encoder and decoder stacks [30]. This architecture was chosen based on the results of prior multi-vendor, multi-disease myocardial segmentation challenges [31]. Leaky rectified linear units (ReLU) were utilized for activation [32], with an instance normalization initializer [33]. An individual nnU-net was optimized for each cardiac view, with the number of output channels determined by the number of cavity and myocardium labels present in each view.

#### Optimization and evaluation

For each cardiac view, an nnU-net network was optimized with a sum of cross-entropy and Dice loss function [34] for a total of 100 epochs using a batch size of 10 and a stochastic gradient descent optimizer with an initial learning rate of 0.01 and Nesterov momentum of 0.99. The learning rate was decayed throughout training following the ‘poly’ learning rate policy [35]. Following training, performance for each network was assessed using Dice scores [36] and Hausdorff distances [37] between predicted and ground truth contours using a single fold validation.

#### Interobserver analysis

To further characterize the performance of the nnU-net segmentations, an interobserver analysis was conducted to determine the variation in myocardial segmentations between two human observers. In this analysis, two expert analysts, each with greater than 10 years of cardiac modeling experience, manually drew contours of the RV and LV myocardium and blood pool at ED and ES for each cardiac view using Segment (Medviso, Lund, Sweden) [29]. This analysis was performed for a subset of 36 cases from the training and validation sets. Dice scores between contours drawn by the two analysts were calculated and compared to the Dice scores achieved by the nnU-net network.

### Automated cardiac shape modeling pipeline testing

The automated cardiac shape modeling pipeline was tested by comparing manually and automatically generated shape models from study participants in the test set. Automatically generated models were first aligned with manually generated models using a rigid registration. Euclidean projection distances were then calculated between points on the automatically generated models and surfaces on the manually generated models, which was the metric used to compute the mean absolute error (MAE) in a global and regional error analysis. Global ventricular measurements were also compared between the manually and automatically generated models by computing LV and RV volumes and masses at ED and ES by numerical integration of mesh volumes. Lastly, manually and automatically generated models were projected onto an ED/ES shape atlas constructed from the shape models in the training/validation set and computed Z-scores were compared.

### Statistical analysis

Statistical analyses were carried out using the SciPy Python library (Python Software Foundation, Wilmington, Delaware, USA; https://www.scipy.org). Summary characteristics of study participants in the training/validation and test sets are reported as mean ± standard deviation or as median (interquartile range), depending on the distribution, for continuous variables and as the count for categorical variables. Normality was tested using Shapiro-Wilks. Differences between these groups were assessed using two-sample *t*-tests or Wilcoxon rank-sum tests, depending on the distribution, for continuous variables and Pearson’s chi-squared tests for categorical variables. The AAFD between predicted and manual labels in the validation set was compared to the AAFD between two manual analyst labels in the validation set using a two-sided t-test. Differences in global ventricular measurements for manually and automatically generated shape models in the test set were assessed using paired-sample t-tests. The distribution of Z-scores for the manually and automatically generated shape models were assessed by a two-sample Kolmogorov–Smirnov test with a significance level of 0.05 and a Holm-Bonferroni correction for multiple comparisons.

## Results

### Individual network performance

#### View classification

Precision, recall, and F1-scores for view classification predictions on the validation set are shown in Table 3. Cardiac views were reliably classified.

**Table 3:** Precision, recall, and F1-scores for cardiac view classification predictions on the validation set

Cardiac view	Precision	Recall	F1-score
2Ch LT	0.88	0.94	0.91
2Ch RT	0.96	0.95	0.96
3Ch	0.38	0.83	0.52
4Ch	0.85	0.92	0.89
LVOT	1.00	0.92	0.96
RVOT	0.78	0.79	0.79
SAx	0.90	0.96	0.93
OTHER	0.97	0.89	0.93

*2Ch LT* two-chamber left, *2Ch RT* two-chamber right, *3Ch* three-chamber, *4Ch* four-chamber, *LVOT* left ventricular outflow tract, *RVOT* right ventricular outflow tract, *SAx* short axis

#### Slice selection

Precision, recall, and F1-scores for slice selection predictions on the validation set are shown in Table 4. SA slices were reliably classified.

**Table 4:** Precision, recall, and F1-scores for short-axis slice selection predictions on the validation set

SAx slice optimality	Precision	Recall	F1-score
Optimal	0.81	0.93	0.86
Non-optimal	0.96	0.87	0.91

*SAx* short axis

#### Phase selection

The AAFD between predicted ES phase labels and manual labels in the validation set is shown in Table 5. The AAFD between two manual analyst labels in the validation set is shown for reference. There was no significant difference between the AAFD between the predicted and manual labels and the AAFD between interobserver labels, as assessed by a two-sided *t*-test with a significance level of 0.05.

**Table 5:** Absolute frame difference (AAFD) between predicted end-systole phase labels and manual labels in the validation set. The AAFD between two manual analyst labels in the validation set is shown for reference

	Predicted vs. Manual	Interobserver	*p*-value
AAFD	1.15 ± 1.02	1.39 ± 1.35	0.18

The AAFD is reported as mean ± standard deviation

#### Anatomical landmark localization

**Fig. 3:**
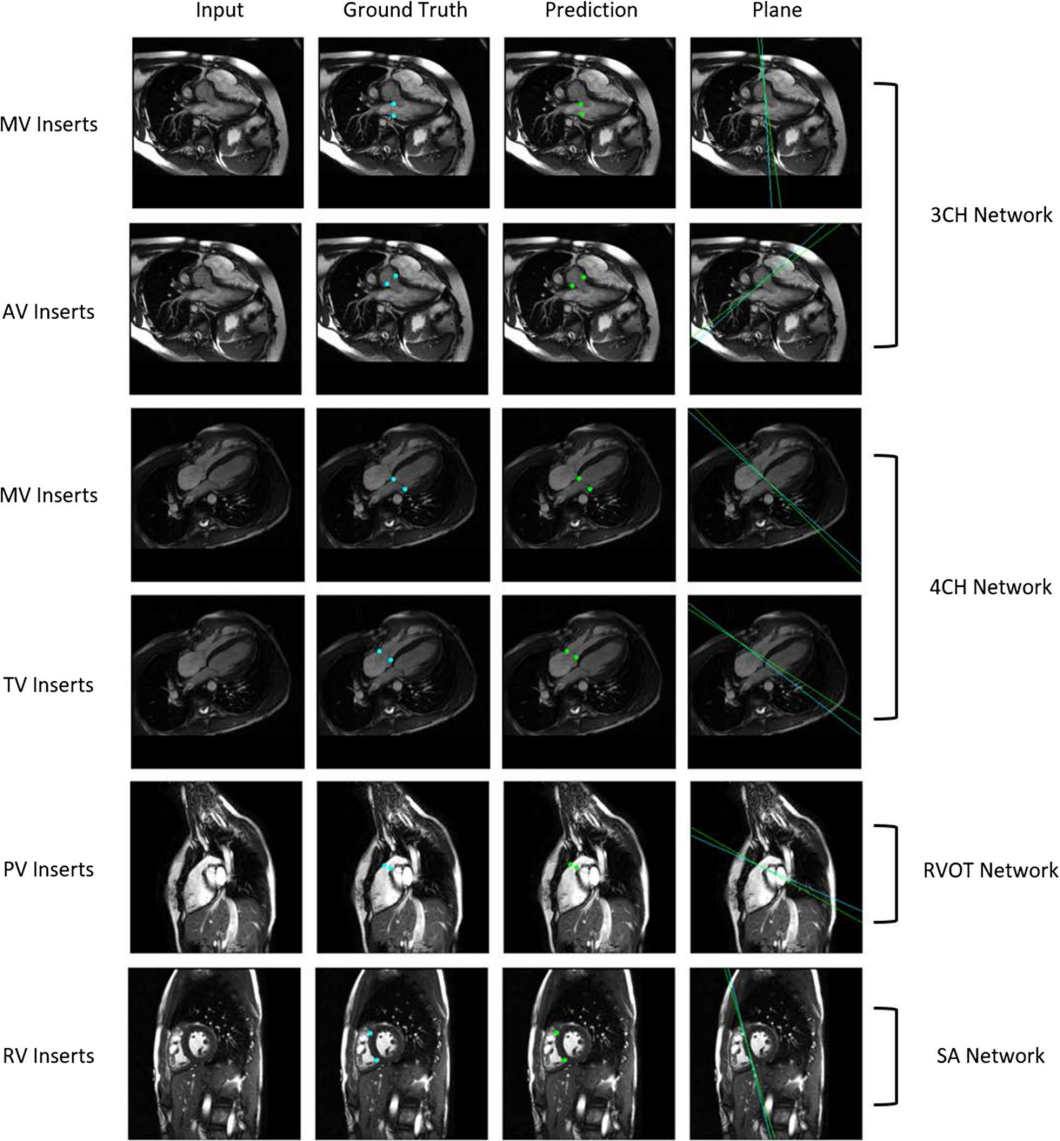
Representative anatomical landmark localization predictions for the 3Ch, 4Ch, RVOT, and SAx views. *3CH* three-chamber, *4Ch* four-chamber, *RVOT* right ventricular outflow tract, *SAx* short axis, *RV* right ventricular, *MV* mitral valve, *AV* aortic valve, *TV* tricuspid valve, *PV* pulmonary valve

Absolute distance errors between predicted and ground truth anatomical landmarks in the validation set are shown in Table 6. For insertion points, the angulation error between predicted and ground truth valve and septal planes is also shown. Representative anatomical landmark localization predictions are shown in Fig. 3. Anatomical landmarks were reliably localized.

**Table 6:** Anatomical landmark localization distance errors and valve and septal plane angulation errors in the validation set

Cardiac view and anatomical landmark	Distance error (mm)	Plane	Angulation error (°)
**3Ch view**			
MV Insert 1	7.1 ± 3.4	MV	14.3 ± 12.2
MV Insert 2	6.4 ± 3.3		
AV Insert 1	10.8 ± 7.5	AV	19.5 ± 17.0
AV Insert 2	9.8 ± 7.5		
**4Ch view**			
MV Insert 1	4.3 ± 2.6	MV	7.1 ± 5.7
MV Insert 2	6.0 ± 3.3		
TV Insert 1	4.9 ± 2.7	TV	10.2 ± 13.1
TV Insert 2	5.0 ± 3.4		
LV Apex	6.2 ± 3.6		
**RVOT view**			
PV Insert 1	13.6 ± 8.1	PV	48.7 ± 37.8
PV Insert 2	17.0 ± 10.4		
**SAx view**			
RV Insert 1	5.9 ± 5.3	Septal	8.7 ± 13.5
RV Insert 2	5.0 ± 3.2		

Distance and angulation errors are reported as mean ± standard deviation. 3Ch: three-chamber; 4Ch: four-chamber; RVOT: right ventricular outflow tract; SAx: short axis; LV: left ventricular; RV: right ventricular; MV: mitral valve; AV: aortic valve; TV: tricuspid valve; PV: pulmonary valve

#### Myocardial image segmentation

**Fig. 4:**
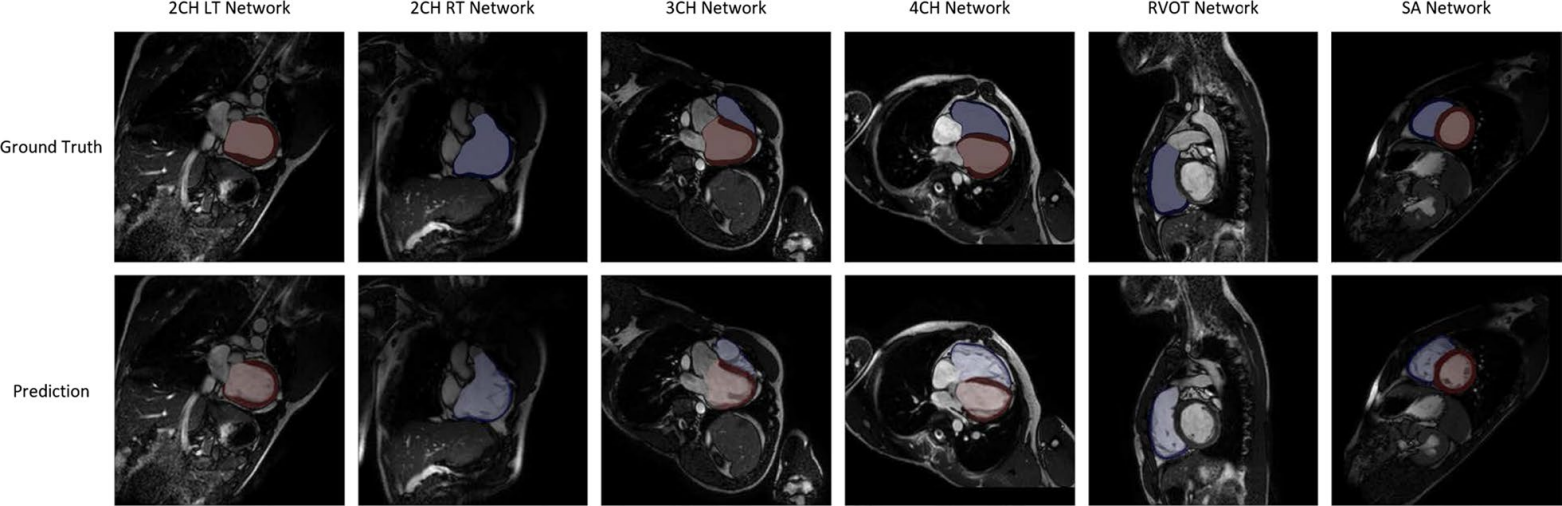
Representative myocardial image segmentation predictions for the 2Ch LT, 2Ch RT, 3Ch, 4Ch, RVOT and SAx views. *2Ch LT* two-chamber left, *2Ch RT* two-chamber right, *3Ch* three-chamber, *4Ch* four-chamber, *RVOT* right ventricular outflow tract, *SAx* short axis

Dice scores and Hausdorff distances between predicted and ground truth contours in the validation set are shown in Table 7. Representative myocardial image segmentation predictions are shown in Fig. 4. Segmentation performance was found to be highly reliable and comparable to the interobserver segmentation error between two expert manual analysts, as shown in Table 8.

**Table 7:** Myocardial image segmentation Dice scores and Hausdorff distances in the validation set

Cardiac view and contour	Dice score	Hausdorff distance (pixels)
2Ch LT view		
LV cavity	0.96 ± 0.02	2.73 ± 1.04
LV myocardium	0.88 ± 0.06	2.94 ± 1.13
2Ch RT view		
RV cavity	0.97 ± 0.02	3.66 ± 2.40
RV myocardium	0.79 ± 0.12	4.88 ± 3.04
3Ch view		
LV cavity	0.96 ± 0.02	3.57 ± 1.68
LV myocardium	0.90 ± 0.03	4.25 ± 3.90
RV cavity	0.95 ± 0.02	2.90 ± 1.95
RV myocardium	0.76 ± 0.09	5.98 ± 6.28
4Ch view		
LV cavity	0.97 ± 0.01	2.57 ± 1.34
LV myocardium	0.90 ± 0.03	4.07 ± 3.12
RV cavity	0.96 ± 0.03	3.60 ± 2.73
RV myocardium	0.77 ± 0.12	3.74 ± 2.06
RVOT view		
RV cavity	0.94 ± 0.03	4.05 ± 2.32
RV myocardium	0.78 ± 0.09	4.25 ± 2.48
SAx view		
LV cavity	0.94 ± 0.03	4.51 ± 3.05
LV myocardium	0.90 ± 0.02	5.40 ± 3.22
RV cavity	0.94 ± 0.02	4.50 ± 2.35
RV myocardium	0.78 ± 0.03	8.62 ± 3.94

Dices scores and Hausdorff distances are reported as mean ± standard deviation. *2Ch LT* two-chamber left, *2Ch RT* two-chamber right, *3Ch* three-chamber, *4Ch* four-chamber, *RVOT* right ventricular outflow tract, *SAx* short axis; *LV* left ventricular, *RV* right ventricular

**Table 8:** Interobserver analysis results showing myocardial image segmentation Dice scores between two expert analysts for a subset of the training and validation sets (n = 36)

Cardiac view and contour	Dice score
LA views	
LV cavity	0.94 ± 0.05
LV myocardium	0.83 ± 0.06
RV cavity	0.91 ± 0.12
RV myocardium	0.54 ± 0.13
SAx view	
LV cavity	0.94 ± 0.08
LV myocardium	0.83 ± 0.07
RV cavity	0.91 ± 0.06
RV myocardium	0.60 ± 0.12

Dices scores are reported as mean ± standard deviation. *LA* long axis, *SAx* short axis, *LV* left ventricular, *RV* right ventricular

### Automated cardiac shape modeling pipeline results

#### Comparison with manual models

**Fig. 5:**
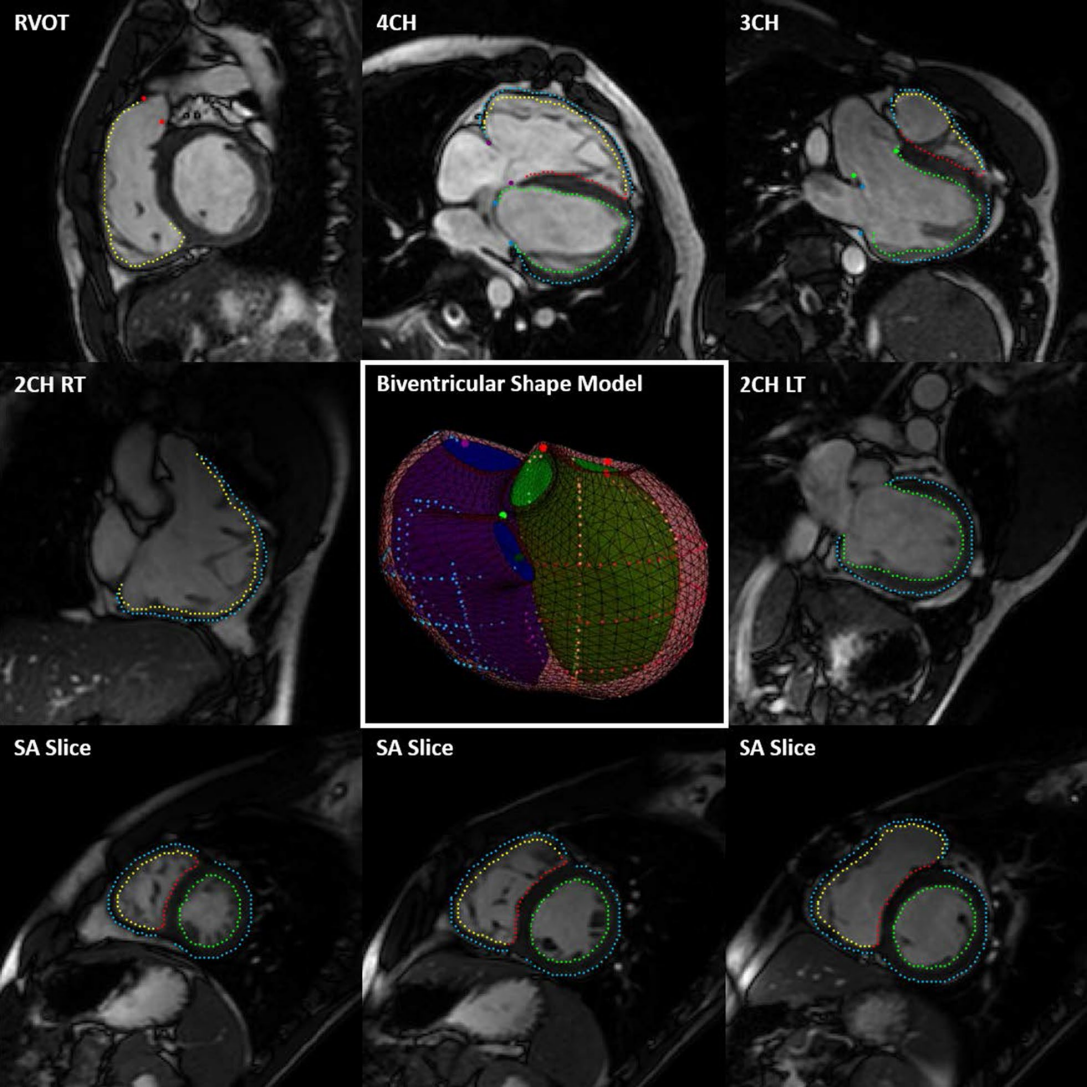
Representative output of the automated cardiac shape modeling pipeline. Extracted contour points for the LV endocardium (green), RV endocardium (yellow), epicardium (cyan), and septum (red) and anatomical landmark points for the MV (blue), AV (green), TV (purple), and PV (red) are shown on corresponding views (outside). The contour points and anatomical landmark points were then fit to a biventricular subdivision surface template mesh resulting in a patient-specific biventricular shape model (center) with surfaces for the LV endocardium (green), RV endocardium (blue), and epicardium (maroon). *2Ch LT* two-chamber left, *2Ch RT* two-chamber right, *3Ch* three-chamber, *4Ch* four-chamber, *RVOT* right ventricular outflow tract; *SAx* short axis, *LV* left ventricular, *RV* right ventricular, *MV* mitral valve, *AV* aortic valve, *TV* tricuspid valve, *PV* pulmonary valve

**Fig. 6:**
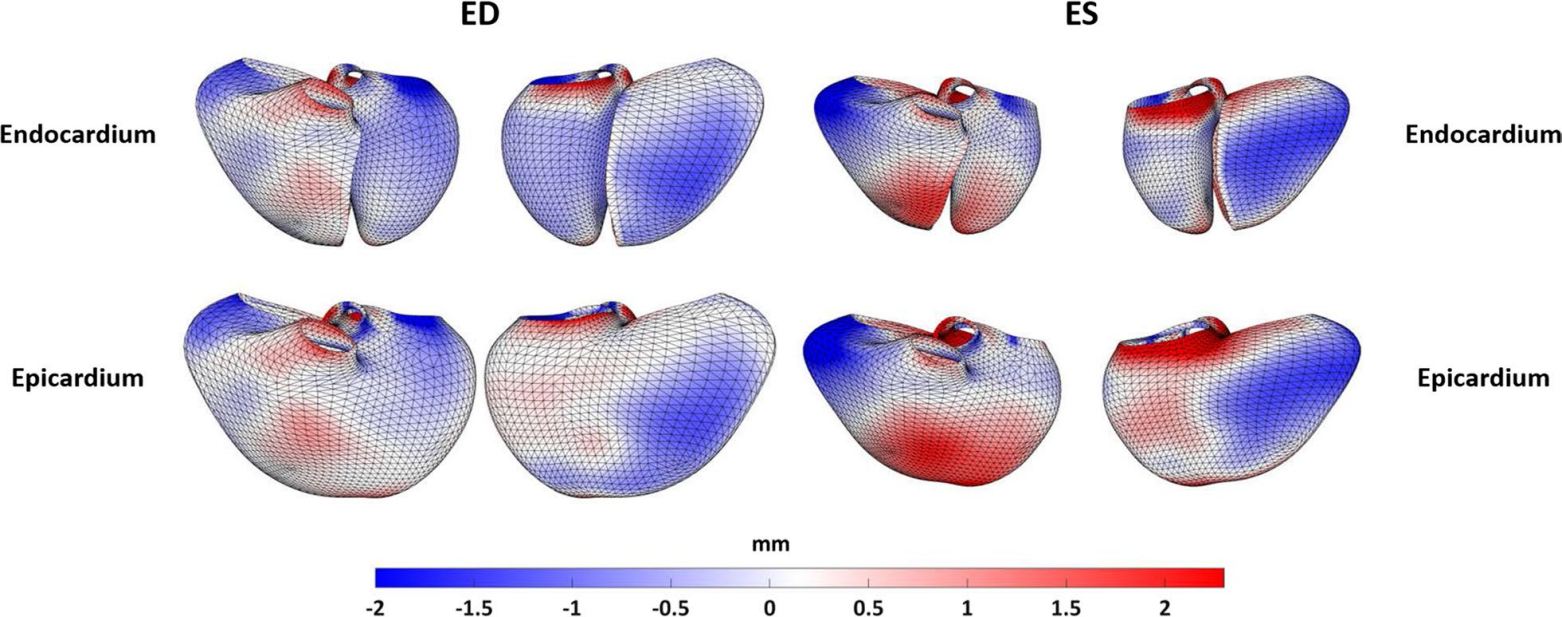
Average inward (blue) and outward (red) Euclidian projection distances between manually and automatically generated shape models in the test set. The range of the color bar accounts for 99% of the observed errors. *ED* end-diastole, *ES* end-systole

**Fig. 7:**
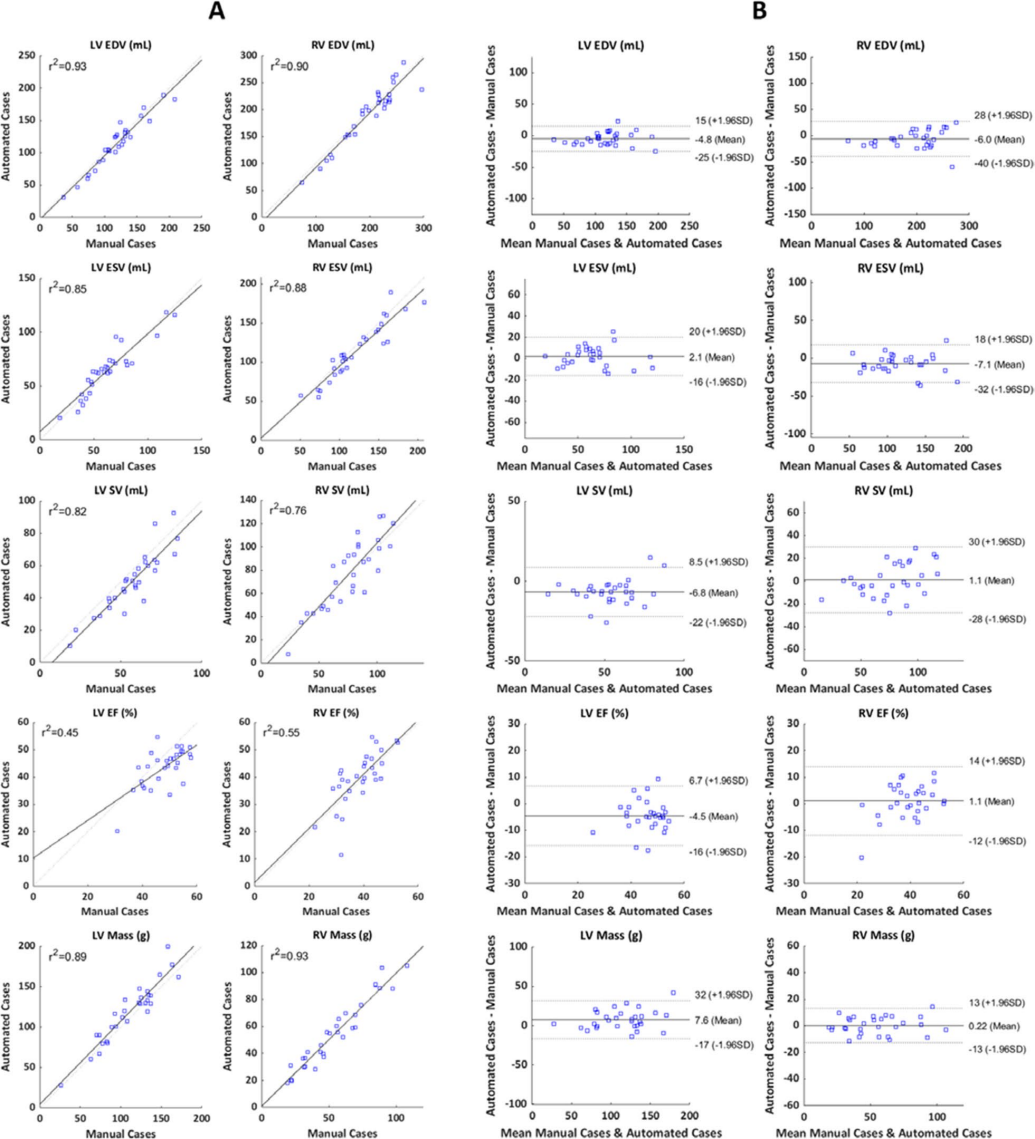
**A** Regression plots showing the correlation between global ventricular measurements for manually and automatically generated shape models in the test set. **B** Bland–Altman plots comparing the correlation of global ventricular measurements for manually and automatically generated shape models in the test set. *LV* left ventricular, *RV* right ventricular, *EDV* end-diastolic volume, *ESV* end-systolic volume, *SV* stroke volume, *EF* ejection fraction

A representative output of the cardiac shape modeling pipeline is shown in Fig. 5, which depicts the myocardial contours and anatomical landmark points that are generated for each cardiac view that are then fit to a subdivision surface template mesh to build a three-dimensional, biventricular shape model. In order to assess the performance of the automated pipeline, the MAE between manually and automatically generated models in the test set was computed. This was done on a global and regional basis for ED and ES models as shown in Table 9. The overall error of the automated models is within voxel resolution of the original CMR images for ED models and approximately at voxel resolution for ES models (Table 2). In order to assess systematic inward or outward surface displacement of the automated models compared to the manual models, the average algebraic Euclidean projection distance for each coordinate point in the biventricular surface mesh was computed and is shown in Fig. 6. Global ventricular measurements including volume and mass metrics were also compared between manually and automatically generated models in the test set. A summary of the global ventricular measurements computed in manually and automatically generated models is shown in Table 10, along with the differences and correlations. Figure 7a shows regression plots and Fig. 7b shows Bland–Altman plots between global ventricular measurements for manually and automatically generated models.

**Table 9:** MAE between manually and automatically generated shape models in the test set based on projection distance

MAE (mm)	ED	ES
Global	1.9 ± 0.5	2.1 ± 0.7
Region		
LV Endocardium	1.6 ± 0.7	1.8 ± 1.0
RV Endocardium	1.8 ± 0.5	2.1 ± 0.5
Septum	1.3 ± 0.4	1.4 ± 0.5
Epicardium	2.2 ± 0.8	2.5 ± 1.0
MV	4.6 ± 0.5	5.6 ± 0.7
AV	5.2 ± 0.1	5.4 ± 0.3
TV	3.6 ± 0.6	3.9 ± 0.5
PV	3.1 ± 0.3	3.0 ± 0.3

Numerical data are reported as mean ± standard deviation. *MAE* mean absolute error, *ED* end-diastole, *ES* end-systole, *LV* left ventricular, *RV* right ventricular, *MV* mitral valve, *AV* aortic valve, *TV* tricuspid valve, *PV* pulmonary valve

**Table 10:** Average global ventricular measurements for manually and automatically generated shape models in the test set as well as differences and correlations

Measure	Manual Cases	Automated Cases	Difference (%)	*R*^2^	*p*-value
LV EDV (mL)	119 ± 36	114 ± 37	− 5 ± 10 (− 4)	0.93	< 0.05
LV ESV (mL)	62 ± 24	64 ± 23	2 ± 9 (3)	0.85	0.26
LV SV (mL)	57 ± 16	50 ± 18	− 7 ± 8 (− 12)	0.82	< 0.01
LV EF (%)	48 ± 7	44 ± 7	− 5 ± 6 (− 9)	0.45	< 0.01
LV Mass (g)	111 ± 33	118 ± 37	8 ± 12 (7)	0.89	< 0.01
RV EDV (mL)	197 ± 51	191 ± 54	− 6 ± 17 (− 3)	0.90	0.07
RV ESV (mL)	121 ± 37	114 ± 36	− 7 ± 13 (− 6)	0.88	< 0.01
RV SV (mL)	76 ± 23	77 ± 29	1 ± 15 (1)	0.76	0.67
RV EF (%)	39 ± 7	40 ± 10	1 ± 7 (3)	0.55	0.39
RV Mass (g)	53 ± 24	54 ± 25	0 ± 7 (0.4)	0.93	0.83

Numerical data are reported as mean ± standard deviation. Differences between the manual and automated cases were assessed using paired-sample t-tests. *LV* left ventricular, *RV* right ventricular, *EDV* end-diastolic volume, *ESV* end-systolic volume; *SV* stroke volume, *EF* ejection fraction

#### Pipeline timing and manual intervention requirements

For a subset of the test set (n = 12), the time required to generate cardiac shape models using the automated pipeline was recorded. Statistics were recorded at multiple institutions for multiple users. Shape models were generated in 5.1 ± 2.8 min on average per model (range 2.5 – 10.2 min). This represents a significant time savings over manual approaches, which typically take 60–90 min on average for a single model. For this subset of cases, the number of times manual override was required was also recorded. The automated pipeline was designed so the user could manually override the automated predictions at each step if necessary. Manual override was only required during the landmark localization step, with interventions occurring for 11.4% of landmarks. The most frequently corrected predictions were for the aortic valve insertions (40% of corrections) and pulmonary valve insertions (40% of corrections). A summary of the necessary manual overrides can be seen in Table 11.

**Table 11:** Occurrence of manual overrides for landmark localization predictions when using the automated pipeline for a subset of the test set (n = 6 internal cases, n = 6 external cases)

Landmark	Manual overrides (%)
AV Inserts	16 of 48 (33)
PV Inserts	16 of 48 (33)
MV Inserts	8 of 96 (8)
RV Inserts	0 of 148)
LV Apex	0 of 12 (0)
Total	40 of 352 (11.4)

Occurrences are reported as n (%). *RV* right ventricular, *LV* left ventricular, *AV* aortic valve, *MV* mitral valve, *PV* pulmonary valve

#### Evaluation of useability for statistical shape modeling

**Fig. 8:**
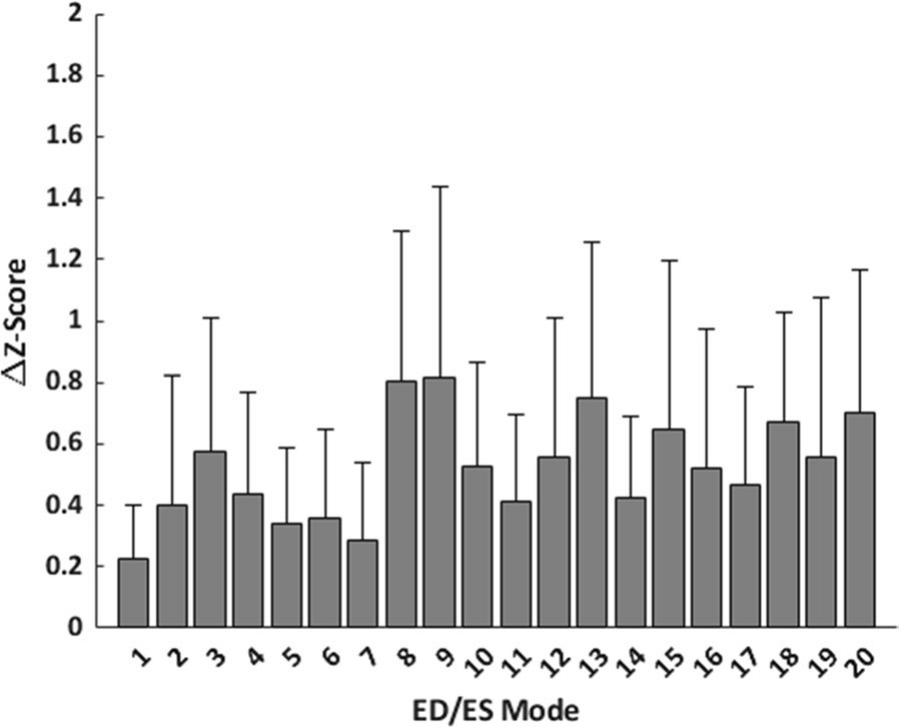
Z-score difference between manually and automatically generated shape models in the test set projected onto an ED/ES shape atlas constructed from shape models in the training/validation set. Bars show the average absolute difference in Z-score, and error bars show the standard deviation

In order to assess the robustness of the automated cardiac shape modeling pipeline for statistical shape modeling applications, the manually and automatically generated models in the test set were projected onto an ED/ES shape atlas derived from shape models in the training/validation set. The mean absolute difference in Z-scores between manually and automatically generated models was then computed for the first 20 modes of the atlas (Fig. 8), which explain approximately 87% of the shape variation in the training/validation set cases. The mean absolute difference in Z-score was below one standard deviation for each of the first 20 modes, and the average mean absolute difference in Z-score for the first 20 modes was 0.5 standard deviations. The distribution of Z-scores for the manually and automatically generated models were not significantly different for each of the first 20 modes, except mode 8, as assessed by a two-sample Kolmogorov–Smirnov test with a significance level of 0.05 and a Holm-Bonferroni correction for multiple comparisons.

## Discussion

In this study, we demonstrate the use of deep learning for automated view classification, slice selection, phase selection, anatomical landmark localization, and myocardial image segmentation that together provide an end-to-end pipeline for cardiac shape modeling. While others have developed automated cardiac shape modeling pipelines [38–41], the pipeline presented herein is the first, to our knowledge, to reliably generate 3D, biventricular shape models, including all four valves, from a raw CMR image dataset for the challenging anatomies seen in rTOF. Overall, the automated pipeline performed well on an independent, multi-institutional test set that included a variety of CMR scanners, including several models that were not included in the training/validation set. (Fig. 2 and Table 2).

The highest errors between the automated and manual models were observed around the valve planes (Table 9 and Fig. 6). This was probably due to the high sensitivity of the fitting of the biventricular subdivision surface template mesh to the location of the valve insertion points, which are extremely sparse compared with the contour points used to fit the LV and RV endocardial and epicardial surfaces. Even with manually generated biventricular shape models, slight deviations in the locations of the valve insertion points can result in significant differences in the valvular anatomy of the fitted models.

With this new automated cardiac shape modeling pipeline, which includes a manual confirmation or override for each step of the workflow, a single cardiac shape model can be made in 5.1 ± 2.8 min on average, whereas manual models generally require 60–90 min per model for an expert analyst. This dramatic reduction in processing time can be useful for estimating global ventricular volumes and masses, for which the automatically generated models demonstrated good agreement with the manual models (Table 10 and Fig. 7). Although differences between automated and manual models reached statistical significance for several global measurements, the magnitude of these differences were small and unlikely to be clinically significant. Moreover, these differences and correlations were similar to previously reported manual interobserver errors and differences between existing clinical techniques, such as the error between CMR and echocardiography [42, 43]. The reduction in processing time can also significantly increase the throughput and clinical translation of more specific atlas-based analyses of biventricular shape. The automatically generated models were able to capture relevant features of regional ED/ES shape variation to within 0.5 standard deviations on average per mode compared with the manually generated models (Fig. 8). With this automated workflow, the analysis of large retrospectively collected datasets, such as the INDICATOR cohort [44], can be rapidly achieved, yielding larger and more comprehensive statistical atlases for shape, biomechanics, and electrophysiology analyses with more statistical power when assessing relationships with independent measures of outcome. Additionally, with an end-to-end pipeline that has processing times more consistent with clinical workflows, the ability to deploy atlas-based analyses in a point-of-care clinical setting to quantify patient-specific anatomy, function, or risk relative to the population would be greatly enhanced.

In the current iteration of the pipeline, the anatomical landmark localization and myocardial image segmentation networks were only trained on cardiac shape models created at ED and ES. This was done because reference manual anatomical landmarks and segmentations for training/validation were only available at ED and ES. This can readily be extended to other timepoints, however, by validating the automated model performance on timepoints throughout the cardiac cycle compared to manual models derived at these same timepoints. Doing so would enable the quantification of dynamic information throughout the cardiac cycle and enable the creation of statistical atlases with much greater temporal resolution. This could assist in the analysis of the effects of ventricular electrophysiologic activation (e.g. bundle branch block, pacing, large scars or patches) on shape and biomechanics. Since the current pipeline was designed as a series of five steps, each of the networks can be improved upon independently of each other. This modularity will be especially useful for extending the automated pipeline to other CHDs with two ventricle morphology, such as coarctation of the aorta, because testing, performance assessment, and any required network retraining will need to be done only on specific steps as needed.

In this study, the ES phase was selected based on the LV cavity in a mid-ventricular SAx slice. For some patients, the presence of right bundle branch blocks or other dyssynchrony may necessitate the selection of independent LV and RV phases. The demonstration of statistical shape modeling presented in this manuscript requires temporal synchronization and the selection of a single ED and ES phase, which may lead to inaccuracies in the RV in the setting of a right bundle branch block. However, the pipeline provides the option of manually selecting independent LV and RV phases, allowing the user to select the option most appropriate for their analyses.

## Limitations

In the current iteration of the pipeline, the anatomical landmark localization and myocardial image segmentation networks were only trained on cardiac shape models created at ED and ES. This was done because reference manual anatomical landmarks and segmentations for training/validation were only available at ED and ES. This can readily be extended to other timepoints, however, by validating the automated model performance on timepoints throughout the cardiac cycle compared to manual models derived at these same timepoints. Doing so would enable the quantification of dynamic information throughout the cardiac cycle and enable the creation of statistical atlases with much greater temporal resolution. This could assist in the analysis of the effects of ventricular electrophysiologic activation (e.g. bundle branch block, pacing, large scars or patches) on shape and biomechanics. Since the current pipeline was designed as a series of five steps, each of the networks can be improved upon independently of each other. This modularity will be especially useful for extending the automated pipeline to other CHDs with two ventricle morphology, such as coarctation of the aorta, because testing, performance assessment, and any required network retraining will need to be done only on specific steps as needed.

In this study, the ES phase was selected based on the LV cavity in a mid-ventricular SAx slice. For some patients, the presence of right bundle branch blocks or other dyssynchrony may necessitate the selection of independent LV and RV phases. The demonstration of statistical shape modeling presented in this manuscript requires temporal synchronization and the selection of a single ED and ES phase, which may lead to inaccuracies in the RV in the setting of a right bundle branch block. However, the pipeline provides the option of manually selecting independent LV and RV phases, allowing the user to select the option most appropriate for their analyses.

## Conclusions

Through the use of deep learning, we were able to automate all of the major steps involved in constructing 3D, biventricular shape models including view classification, slice selection, phase selection, anatomical landmark localization, and myocardial image segmentation. To our knowledge, this is the first fully automated, end-to-end pipeline that can robustly create shape models for the challenging anatomies present in rTOF. With this approach, we can greatly reduce the manual input required to create shape models enabling the rapid analysis of large-scale datasets and the potential to deploy statistical atlas-based analyses in point-of-care clinical settings.

## Data Availability

Training data and networks are available from cardiacatlas.org.

## Abbreviations

2Ch LT: Two-chamber left
2Ch RT: Two-chamber right
3Ch: Three-chamber
3D: Three dimensional
4Ch: Four-chamber
AAFD: Absolute average frame difference
AV: Aortic valve
BSA: Body surface area
CAP: Cardiac Atlas Project
CHD: Congenital heart disease
CMR: Cardiovascular magnetic resonance
CNN: Convolutional neural network
ED: End-diastole
EDV: End-diastolic volume
EF: Ejection fraction
ES: End-systole
ESV: End-systolic volume
FCN: Fully convolutional network
LA: Long axis
LSTM: Long short-term memory
LV: Left ventricle/left ventricular
LVOT: Left ventricular outflow tract
MAE: Mean absolute error
MV: Mitral valve
PV: Pulmonary valve
ReLU: Rectified linear units
RNN: Recurrent neural network
rTOF: Repaired tetralogy of Fallot
RV: Right ventricle/right ventricular
RVOT: Right ventricular outflow tract
SAx: Short axis
SELU: Scaled exponential linear units
SV: Stroke volume
TOF: Tetralogy of Fallot
TV: Tricuspid valve
